# The Treatment of Chronic Nerve Injuries

**Published:** 1904-09-17

**Authors:** 


### THE TREATMENT OF CHRONIC NERVE INJURIES.

The nerves commonly injured, roughly in their order of frequency, are: the median, the ulnar, the musculo-spiral, the external popliteal, the circumflex, and the cords of the brachial plexus. Nerves may be injured in open wounds and completely or partially divided. They may be injured subcutaneously, either by rupture or as the result of pressure suddenly or gradually applied. Mr. Sherren [1] makes the following practical classification:—

I. Anatomical division (section or rupture)—
*a.* Partial; *b.* Complete.

II. Physiological division (compression)—
*a.* Partial; *b.* Complete.

In the case of a wound which implicates a nerve the nerve should be exposed without undue delay, every care being taken to combat wound infection, as suppuration, leading as it does to the formation of dense scar tissue, is *the* great hindrance to complete recovery. If it be found that the nerve is completely divided, the ends must be brought into contact and sutured. Fine catgut is the best material to use, but if there is any tension silk must be employed, as the catgut is liable to yield. If the nerve is partially divided the gap should be sutured, as it is found in practice that if this is not done recovery is often incomplete.

In cases of primary suture the wound must be drained for the first 24 hours. The limb must be placed on a splint with the joints flexed so as to prevent tension on the injured nerve. Before recovery ensues 12 months or more must, as a rule, elapse, and during this time every care must be taken to prevent trophic changes in the parts supplied by the damaged nerve. The nutrition of the skin and muscles must be kept up by systematic massage, employed at least three times a week; in this way trophic sores can usually be avoided, and the skin may retain an almost normal appearance. Electricity, in any form, is unnecessary.

In those cases where operation is performed after a considerable time has elapsed since the injury the nerve ends will have to be freed from surrounding scar tissue before being sutured. Nerves retract very little after division so that the cut ends usually remain close together. Before suturing it is necessary to resect the bulbous end of the upper part of the divided nerve and to take also a small section from the lower part. If the gap left is so great that the divided ends cannot be approximated even when the joints are flexed nerve stretching may be employed: by this means a gap of $1\frac{1}{2}$ inch may be bridged. If there still remains too great a distance between the nerve ends, nerve-grafting may be employed, or, failing this, suture at a distance with catgut threads enclosed in a decalcified bone tube.

Nerve anastomosis should be reserved for those cases in which the upper end of the divided nerve cannot be found. Resection of bone, for the purpose of shortening the limb and so allowing the ends of the nerve to be approximated is not permissible. In nerve-grafting, the method of choice, the graft may be obtained from a recently amputated limb, from an animal—*e.g.* rabbit—or, where the musculo-spiral nerve is being operated on, from the patient's radial nerve of the same side. Owing to the free anastomoses of the radial nerve, its removal as a rule produces no ill results.

In cases of subcutaneous injury, massage is the first indication. If, instead of recovering, the affected muscles waste and the reaction of degeneration develops, the nerve must be cut down upon and examined, and, if bound up with fibrous tissue, the injured part must be excised and the nerve sutured. In such a case the patient must be warned beforehand that the condition of the part will be worse for some time after the operation. When a nerve has been dissected out from callus or fibrous tissue, precautions must be taken to prevent it from contracting fresh adhesions, and this is best done by placing a piece of sterile gold foil between the two structures. Gold foil may be left in the tissues for an indefinite time without giving rise to any irritation. Dr. Sherren advocates that all subcutaneous nerve injuries which give the reaction of degeneration at the end of 10 days should be submitted to operation.

[1] Clinical Jour., Aug. 31.

